# Triglyceride/HDL-Cholesterol Ratio as an Index of Intracranial Atherosclerosis in Nonstroke Individuals

**DOI:** 10.3389/fneur.2020.504219

**Published:** 2021-01-18

**Authors:** Min-Hee Woo, Kee Ook Lee, Darda Chung, Jung-Won Choi, Sang-Heum Kim, Seung-Hun Oh

**Affiliations:** ^1^Department of Neurology, CHA Bundang Medical Center, CHA University, Seongnam, South Korea; ^2^Department of Radiology, CHA Bundang Medical Center, CHA University, Seongnam, South Korea

**Keywords:** triglyceride, high-density lipoprotein, intracranial atherosclerosis, large artery atherosclerosis, small vessel disease, hyperlipidemia

## Abstract

**Background:** Triglyceride (TG)/high-density lipoprotein cholesterol ratio (THR) is a marker of dyslipidemia, and high THR is associated with an increase in cardiovascular events. In the present study, whether THR was associated with various markers of cerebral vascular pathologies, atherosclerosis of major cerebral arteries, including large artery atherosclerosis (LAA) and cerebral small vessel disease (SVD), in neurologically healthy individuals was investigated.

**Methods:** Vascular risk factors, brain magnetic resonance imaging (MRI) scans, and MR angiograms of 851 study subjects were evaluated. Findings of extracranial atherosclerosis (ECAS) and intracranial atherosclerosis (ICAS) were considered indices of LAA based on brain MR angiograms. The presence of silent lacunar infarct (SLI) and white matter hyperintensities (WMHs) were evaluated as indices of SVD based on brain MRIs.

**Results:** Subjects with ICAS (odds ratio, 1.83; 95% confidence interval, 1.06–3.16; *P* = 0.03) were significantly more likely to have high THR tertile (THR > 2.06) than low THR tertile (THR < 1.37) after adjusting for cardiovascular risk factors. THR was higher in subjects with multiple ICAS lesions than in those with single ECAS or without ICAS lesions. Associations among THR tertiles in ECAS, SLI, and WMHs were not significant.

**Conclusion:** In the present study, a positive association between high THR and the development of ICAS was observed in neurologically healthy participants.

## Introduction

Modification of vascular risk factors is important for future stroke prevention. Ischemic stroke is caused by heterogeneous vascular pathologies in the brain (1). Atherosclerosis of major cerebral arteries is one of the strongest risk factors for ischemic stroke. The large artery atherosclerosis (LAA) is divided into extracranial atherosclerosis (ECAS) and intracranial atherosclerosis (ICAS) based on the anatomical site of atherosclerosis. In recent studies, ECAS and ICAS were reported to differ based on ethnic background and pathogenesis (2–4).

Cerebral small vessel diseases (SVDs), such as silent lacunar infarct (SLI) and cerebral white matter hyperintensities (WMHs), are other risk factors for ischemic stroke (5). SVD is caused by occlusion of small perforating arteries or arterioles in the brain (6). Although SVDs share common risk factors with LAA, the pathogenesis is distinct from LAA (7). These findings indicate novel factors besides classical vascular risk factors for ischemic stroke may be involved in the distinct break cerebrovascular pathology.

Hyperlipidemia, usually defined as high level of serum low-density lipoprotein cholesterol (LDL-C), is a risk factor for coronary arterial atherosclerotic disease. Statins can significantly decrease (up to 37%) the incidence of cardiovascular disease (8). Serum LDL-C levels are also a risk factor for ischemic stroke. The Stroke Prevention by Aggressive Reduction in Cholesterol Levels trial in patients with recent stroke or transient ischemic attack clearly showed that intensive lowering of LDL-C using statin treatment reduced the overall incidence of strokes and cardiovascular events (9). However, residual cardiovascular risk remains high despite significant effects of LDL-C lowering therapy for prevention of ischemic stroke. Because many patients with ischemic stroke still have a high residual risk of recurrent stroke or cardiovascular events despite extensive LDL-C lowering treatment, additional lipid modifications beyond controlling traditional vascular risk factors are needed to reduce the residual risk.

Triglyceride (TG) and high-density lipoprotein (HDL-C) are lipid elements that are commonly tested in clinical setting. Several studies have shown that high level of TG contributes to the development and progression of atherosclerosis and risk for ischemic stroke (10–12). Low level of HDL-C increases the rate of ischemic stroke (13) and carotid stenosis (14).

Recently, the LDL particle phenotype has attracted more attention than the total amount of LDL-C because small, dense LDL particles are highly atherogenic. Atherogenic dyslipidemia, defined as high level of serum TG, high level of small/dense LDL particles, and low level of HDL-C, evidently increases cardiovascular disease (15). Furthermore, the simultaneous use of TG and HDL-C has been found to be more useful than isolated lipid levels because this level closely reflects the complex interactions of lipoprotein metabolism (16). The TG and HDL-C level ratio (THR) was shown directly correlated with small, dense LDL particles in several studies, indicating THR could be feasibly used as an index of atherogenic dyslipidemia in the clinical setting (17–19). In addition, high THR is associated with insulin resistance, metabolic diseases, and cardiocerebrovascular diseases (20–22).

However, whether THR is associated with different cerebrovascular pathologies remains unknown. In several studies, a positive association was observed between THR and LAA (23, 24); however, other research has shown an association between THR and SVDs (25). Moreover, the association of THR with four distinct cerebrovascular pathologies (ECAS, ICAS, SLI, and WMH) in a single cohort has not been investigated. In the present study, the association of THR with different cerebrovascular pathologies in nonstroke individuals was investigated.

## Patients and Methods

### Study Population and Protocol

The study was a retrospective analysis of neurologically healthy individuals who visited the outpatient clinic of the Department of Neurology or Healthcare Center in the CHA Bundang Medical Center between March 2008 and December 2014. All subjects presented for routine health examinations or medical attention due to underlying cardiovascular risk factors. Briefly, only individuals ranging from 50 to 79 years of age who underwent brain magnetic resonance imaging (MRI) and magnetic resonance angiography (MRA) were enrolled. The medical records, laboratory tests results, and radiological findings were reviewed. The study included only subjects whose records contained adequate demographic, radiological, and laboratory data. Among 959 study subjects, 108 were excluded for the following reasons: inadequate medical information (*n* = 22), no laboratory tests performed (*n* = 74), and previous history of neurological disease (*n* = 12). A total of 851 subjects were included in this study. Each subject's data were deidentified prior to analysis. The institutional review board (IRB) of CHA Bundang Medical Center approved the study (IRB no. BD-2010-083).

### Diagnostic Criteria for Clinical Characteristics

Hypertension was diagnosed if the subject had a systolic blood pressure ≥ 140 mm Hg or a diastolic blood pressure ≥ 90 mm Hg on repeated measurements, or the subject was taking antihypertensive medication. Diabetes mellitus was diagnosed if the subject had a fasting plasma glucose ≥ 7.0 mmol/L or was taking antidiabetic medications or insulin. Hypercholesterolemia was diagnosed if the subject had a total cholesterol ≥ 5.69 mmol/L or was taking lipid-lowering agents. Current smoking was defined if the subject had smoked within 1 year prior to examination. Coronary arterial occlusive disease (CAOD) was diagnosed if subjects had a history of acute myocardial infarction, unstable angina, CAOD confirmed by angiography, or coronary surgery or intervention. Chronic kidney disease (CKD) was defined as an estimated glomerular filtration rate <60 mL/min/1.73 m^2^.

### Measurement of Blood Parameters and THR

Laboratory data collected for analysis included serum fasting glucose, total cholesterol, HDL-C, and TG. The THR was calculated as TG divided by HDL-C. To evaluate the factors associated with THR, subjects were divided into three groups based on serum THR tertile: (1) low THR tertile group (T1), THR < 1.37; (2) middle THR tertile group (T2), THR ranging from 1.37 to 2.06; and (3) high THR tertile group (T3), THR > 2.06.

### Diagnostic Criteria for Cerebral Atherosclerosis and Cerebral SVDs

Brain MRI and MRA were performed using one of three 1.5-T MR systems (Sonata, Siemens Healthcare, *n* = 568; Signa Excite, GE Healthcare, *n* = 88; Signa HDx, GE Healthcare, *n* = 95). A radiologist who was blinded to clinical and laboratory data assessed the images. ECAS and ICAS were evaluated as MR indices of LAA based on the location of atherosclerotic lesions visualized using MRA. The ECAS was defined as stenosis ≥50% in the external cranial portion of the internal carotid artery or vertebral artery on gadolinium contrast-enhanced MRA, using methods described in the North American Symptomatic Carotid Endarterectomy Trial study (26). The ICAS was defined as stenosis ≥50% in the proximal portions of the middle cerebral artery, anterior cerebral artery, posterior cerebral artery, intracranial portion of the vertebral artery, and basilar artery based on time-of-flight image, using the method described in the Warfarin vs. Aspirin for Symptomatic Intracranial Disease study (27). Normal arterial variations, such as a unilateral origination of the bilateral anterior cerebral arteries or fetal-type posterior cerebral artery, were not regarded as true atherosclerosis.

Next, SLI and WMHs were evaluated as MR indices of SVD visualized on brain MRI. The SLI was defined as a small (3–15 mm in diameter) cavity-like lesion in an area of low signal intensity on T1-weighted images [repetition time (TR)/echo time (TE) = 560/14 ms]. The WMH was defined as a hyperintense lesion in an area of bilateral cerebral white matter visualized on a fluid-attenuated inversion recovery image (TR/TE = 9,000/105 ms, inversion time, 2,500 ms). The degree of the WMH was scored using the visual grading method proposed by Fazekas et al. (28). Periventricular and deep subcortical white matter scores were added (ranging from 0 to 6 points), and lesions with a score ≥3 points were regarded as WMHs in the brain.

### Statistical Analyses

To evaluate the factors associated with THR tertile group, a between-group comparison was conducted using analysis of variance (ANOVA) for continuous variables and χ^2^ test for categorical variables. To evaluate the independent association of ECAS, ICAS, SLI, or WMH across THR tertile groups, binary logistic regression analyses were performed between each group with the low THR tertile group as a reference group. Potential confounding factors were adjusted including age, gender, hypertension, diabetes, statin medication, current smoking, CAOD, CKD, and other significant variables obtained from univariate analysis. Odds ratio (OR) and 95% confidence interval (CI) were calculated. All statistical analyses were performed using the R package for Windows (version 3.1.3; R Foundation for Statistical Computing, Vienna, Austria). A two-sided *P* < 0.05 was considered statistically significant.

## Results

The mean age of the 851 study subjects was 65.2 ± 8.8 years, and 60.4% were females. The mean THR was 1.5 ± 1.5. The prevalence of ECAS, ICAS, SLI, and WMHs was 14.2, 13.6, 16.6, and 30.6%, respectively. The distributions of several baseline characteristics of the entire study population across THR tertiles are shown in Table 1. The prevalence of ICAS was significantly different among THR tertile groups, with a higher THR associated with a more frequent prevalence of ICAS (*P* = 0.048, Table 1). The prevalence of ECAS, SLI, and WMHs did not differ among THR tertile groups. Logistic regression analyses were performed to compare ECAS, ICAS, SLI, and WMHs across THR tertile groups after adjusting for confounding factors (age, gender, hypertension, diabetes mellitus, smoking status, statin medication, CAOD, and CKD; Table 2). The prevalence of ICAS was significantly higher in the high THR group (T3) than in the low THR tertile group (T1) (OR, 1.83; 95% CI, 1.06–3.16; *P* = 0.03). The prevalence of ICAS was not significantly different between the middle and low THR tertile groups. The prevalence of ECAS, SLI, and WMHs did not differ among THR tertile groups based on multivariate logistic regression analysis.

**Table 1:** Clinical characteristics of subjects based on THR tertiles.

	**ALL**	**T1**	**T2**	**T3**	***P* value**
	**(*n* = 851)**	**(*n* = 283)**	**(*n* = 284)**	**(*n* = 284)**	
Gender (female, %)	514 (60.4)	190 (67.1)	180 (63.4)	144 (50.7)	<0.001
Age, years	65.2 ± 8.8	65.7 ± 9.1	65.7 ± 9.1	64.2 ± 8.1	0.072
Hypertension (%)	470 (55.2)	136 (48.1)	159 (56.0)	175 (61.6)	0.005
Diabetes mellitus (%)	209 (24.6)	58 (20.5)	66 (23.2)	85 (29.9)	0.027
Hyperlipidemia (%)	235 (27.6)	63 (22.3)	70 (24.6)	102 (35.9)	0.001
Current smoking status (%)	158 (18.6)	41 (14.5)	54 (19.0)	63 (22.2)	0.061
CAOD (%)	62 (7.3)	17 (6.0)	22 (7.7)	23 (8.1)	0.591
CKD (%)	159 (18.8)	45 (16.0)	49 (17.4)	65 (23.0)	0.077
Statin medication (%)	174 (20.4)	47 (16.6)	51 (18.0)	76 (26.8)	0.005
SBP, mm Hg	131.0 ± 18.9	130.7 ± 20.6	130.9 ± 20.0	131.6 ± 15.6	0.842
DBP, mm Hg	80.0 ± 11.5	79.6 ± 12.4	79.8 ± 11.6	80.6 ± 10.6	0.562
Glucose, mmol/L	7.6 ± 2.9	7.6 ± 2.9	7.5 ± 2.7	7.7 ± 3.0	0.650
eGFR, mL/min/1.73 m^2^	75.0 ± 19.6	75.5 ± 18.6	76.4 ± 20.2	73.2 ± 19.8	0.139
Total cholesterol, mmol/L	4.9 ± 1.1	4.7 ± 1.0	4.9 ± 1.0	5.0 ± 1.3	0.002
HDL-C, mmol/L	1.2 ± 0.3	1.5 ± 0.3	1.2 ± 0.2	1.0 ± 0.2	<0.001
TG, mmol/L	1.7 ± 1.2	0.8 ± 0.2	1.4 ± 0.3	2.8 ± 1.5	<0.001
**LAA**					
ECAS (%)	121 (14.2)	36 (12.7)	40 (14.1)	45 (15.9)	0.554
ICAS (%)	116 (13.6)	28 (9.9)	40 (14.1)	48 (17.0)	0.048
**SVD**					
SLI (%)	141 (16.6)	48 (17.0)	43 (15.1)	50 (17.6)	0.715
WMH (%)	260 (30.6)	90 (31.8)	791 (27.8)	91 (32.0)	0.471

*Values are presented as percentages or means ± standard deviation. P values were derived from an analysis of variance (ANOVA) across THR tertile groups. T1, T2, and T3 represent low, middle, and high THR tertile groups, respectively. THR, TG/HDL-C ratio; CAOD, coronary arterial occlusive disease; CKD, chronic kidney disease; SBP, systolic blood pressure; DBP, diastolic blood pressure; eGFR, estimated glomerular filtration rate; HDL-C, high-density lipoprotein cholesterol; TG, triglyceride; LAA, atherosclerosis of major cerebral arteries; SVD, small vessel disease; ECAS, extracranial atherosclerosis; ICAS, intracranial atherosclerosis; SLI, silent lacunar infarct; WMH, white matter hyperintensity; OR, odds ratio; CI, confidence interval*.

**Table 2:** Logistic regression analysis of ECAS, ICAS, SLI, and WMH based on THR tertiles.

	**ECAS**		**ICAS**		**SLI**		**WMH**	
	**OR (95% CI)**	***P***	**OR (95% CI)**	***P***	**OR (95% CI)**	***P***	**OR (95% CI)**	***P***
T1	Ref	—	Ref	—	Ref	—	Ref	—
T2	1.04 (0.63–1.71)	0.883	1.44 (0.85–2.46)	0.177	0.83 (0.52–1.34)	0.450	0.77 (0.51–1.15)	0.202
T3	1.24 (0.74–2.06)	0.411	1.83 (1.06–3.16)	0.030	0.96 (0.59–1.56)	0.868	1.03 (0.69–1.55)	0.833

*The analyses were conducted after adjusting age, gender, hypertension, diabetes mellitus, smoking status, statin medication, CAOD, and CKD*.

*T1, T2, and T3 represent low, middle, and high THR tertile groups, respectively*.

*Ref represents the low THR tertile group (T1) as a reference group for analyses*.

*THR, TG/HDL-C ratio; ECAS, extracranial atherosclerosis; ICAS, intracranial atherosclerosis; SLI, silent lacunar infarct; WMH, white matter hyperintensity; OR, odds ratio; CI, confidence interval; CAOD, coronary arterial occlusive disease; CKD, chronic kidney disease*.

In the logistic regression analysis to evaluate the individual risk factors associated with LAA and SVD indices, a significant association was observed between THR and ICAS (OR, 1.24; 95% CI, 1.10–1.39; *P* = 0.001). Other significant variables for ICAS were older age and high prevalence of hypertension. THR showed no significant association in subjects with ECAS, SLI, and WMHs. Significant variable for ECAS was high prevalence of diabetes mellitus. Significant variables for SLI were older age, a high prevalence of hypertension, and current smoker. Significant variables for WMH were older age, a high prevalence of hypertension, and CKD (Table 3).

**Table 3:** Logistic regression analysis of vascular risk factors for ECAS, ICAS, SLI, and WMH.

	**ECAS**		**ICAS**		**SLI**		**WMH**	
	**OR (95% CI)**	***P***	**OR (95% CI)**	***P***	**OR (95% CI)**	***P***	**OR (95% CI)**	***P***
Gender, female	1.09 (0.68–1.75)	0.711	1.15 (0.71–1.88)	0.573	0.77 (0.49–1.21)	0.257	1.15 (0.79–1.69)	0.465
Age	1.43 (0.93–2.19)	0.103	2.12 (1.34–3.36)	0.001	2.64 (1.71–4.06)	<0.001	4.95 (3.44–7.11)	<0.001
Hypertension	1.37 (0.90–2.11)	0.147	1.92 (1.21–3.05)	0.006	2.12 (1.38–3.26)	0.001	1.74 (1.24–2.45)	0.001
Diabetes mellitus	1.80 (1.17–2.77)	0.007	1.10 (0.69–1.75)	0.685	1.15 (0.75–1.76)	0.521	1.04 (0.72–1.51)	0.839
Current smoking	1.15 (0.65–2.02)	0.632	1.56 (0.89–2.73)	0.123	1.84 (1.11–3.06)	0.018	1.33 (0.83–2.12)	0.232
CAOD	1.21 (0.61–2.40)	0.584	1.84 (0.96–3.52)	0.066	0.69 (0.33–1.43)	0.317	0.89 (0.50–1.59)	0.695
CKD	1.24 (0.76–2.02)	0.395	1.24 (0.76–2.03)	0.395	1.55 (0.99–2.44)	0.057	1.90 (1.28–2.81)	0.001
THR	1.06 (0.95–1.19)	0.314	1.24 (1.10–1.39)	0.001	1.07 (0.96–1.25)	0.140	1.00 (0.90–1.11)	0.982

*CAOD, coronary arterial occlusive disease; CKD, chronic kidney disease; ECAS, extracranial atherosclerosis; ICAS, intracranial atherosclerosis; SLI, silent lacunar infarct; WMH, white matter hyperintensity; THR, triglyceride (TG)/high-density lipoprotein cholesterol (HDL-C) ratio; OR, odds ratio; CI, confidence interval*.

**Figure 1:**
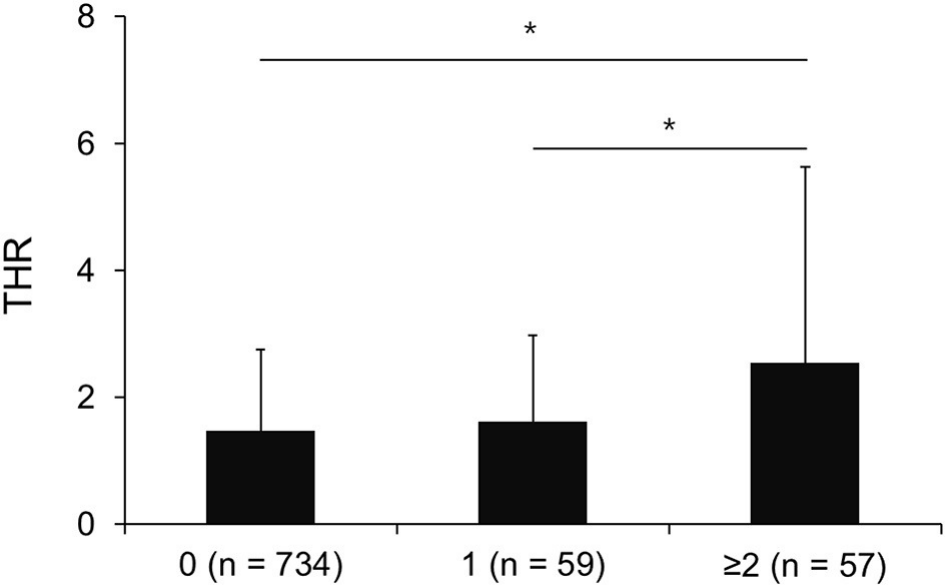
Mean THR among patients without ICAS lesion (*n* = 734), patients with single ICAS lesion (*n* = 59), and patients with multiple ICAS lesions (*n* = 57). The *X* axis represents the number of ICAS lesions. * <0.05 based on Tukey *b* test. The error bar represents the standard deviation.

The effects of THR on the number of ICAS lesions were investigated in patients without ICAS lesion (*n* = 734), patients with single ICAS lesion (*n* = 59), and patients with multiple ICAS lesions (*n* = 57). Statistical significant difference was observed in the THR among the three groups based on ANOVA (*F* = 14.03, *P* < 0.001). The *post hoc* analysis showed that subjects with multiple ICAS lesions had higher THR (2.55 ± 3.09) than subjects without ICAS lesions (1.47 ± 1.29) and subjects with a single ICAS lesion (1.61 ± 1.37; Figure 1).

## Discussion

In the present study, THR was closely associated with ICAS. After adjusting for confounding factors, subjects with high THR tertile displayed 83% higher prevalence of ICAS compared with subjects with low THR tertile. Conversely, THR was not associated with other cerebrovascular pathologies including ECAS and SVD. In agreement with our findings, Sirimarco et al. reported that symptomatic ICAS was strongly associated with low HDL-C and high TG levels (24). In addition, Kang et al. demonstrated that THR was associated with the presence of a steno-occlusive lesion in the basilar artery (23). These results indicate THR as an index of atherogenic dyslipidemia is a useful marker for ICAS, and different pathogenesis exists between ICAS and other cerebrovascular pathologies.

Although the mechanism of the differential impact of THR on ICAS and ECAS is unclear, several explanations are possible. In recent studies, EC and IC arteries were shown to have different metabolic and biological properties. The IC arteries have higher antioxidant capacity and become more prone to oxidative stress than EC arteries (29). HDL-C has antioxidant activity, which inhibits the oxidation of phospholipids and the activity of LDL-C (30). Low HDL-C possibly increases the oxidative stress in the more vulnerable IC arteries than EC arteries, which leads to development of ICAS. Another explanation is the closed link between metabolic syndrome (MetS) and ICAS. In several studies, MetS was more specifically associated with ICAS than ECAS (31–33). Because both TG and HDL-C are indicative of MetS, high THR may contribute to development of ICAS. In addition, MetS is associated with insulin resistance and increases the patient's vulnerability to oxidative stress (34). Insulin resistance was also associated with ICAS in nondiabetic patients (35). TG and HDL-C are independently associated with insulin resistance (36) but are more likely to be caused by abnormalities unrelated to insulin resistance (37). Therefore, THR may be a physiologically more relevant choice than HDL-C or TG alone. In addition, elevated TG and reduced HDL-C levels facilitate the formation of atherogenic LDL particles (19). Because of pathophysiological characteristics of both adventitia and media of the intracranial arteries, small, dense LDL particles penetrate the arterial wall more easily and bind to proteoglycans, rendering the arteries more susceptible to oxidative modifications (24, 38). Overall, the combination of these two conditions (elevated TG and reduced HDL-C levels) may be specifically associated with ICAS and higher risk of early recurrent stroke (24).

In addition, THR was not associated with SLI and WMHs as indices of SVD in the present study. To date, few studies have been performed within the context of observing an association between THR and SVD. In a cross-sectional study in a healthy Korean population, a positive association was observed between THR and males with silent brain infarct (25). In our population, no associations were found among THR tertiles in SLI and WMHs. Our main finding is that THR and ICAS are closely related to positive correlation. Although low HDL-C level was shown associated with WMH severity (39), the authors did not evaluate the relationship between THR and WMH. Reasons for discrepancies between previous studies and our data may be due to differences in study design and subject characteristics. In contrast to the well-known association between hyperlipidemia and LAA, the relationship between hyperlipidemia and SVD is unclear (7). SVDs were shown to have mechanistically different pathogenesis than LAA, such as lipohyalinosis, segmental demyelination, and endothelial dysfunction, indicating the nonatherogenic nature of SVDs (40). Because of the current lack of data, further studies are needed to clarify the exact association between THR and SVD.

The present study had several limitations. First, because this study was retrospective in design, a selection bias existed. Furthermore, the demographic characteristics were not the same as the general population because the study subjects visited the outpatient department for seeking underlying cardiovascular risk factors. For this reason, the possibility of selection bias was not completely excluded. Second, all study subjects were Koreans, so the results cannot be generalized to the general population. Because the prevalence of ICAS and SVD is higher in Asians than in the Western population, the results may differ across ethnicities. Third, the definition in the present study of atherosclerosis based on brain MRA did not include mild cases because of low MRA resolution. Fourth, this is a single-center study, and the sample size was too small to be generalized, requiring large-scale external validation of the current study. Fifth, statin was included in this study, but antiplatelet or anticoagulant agents were not included in the analysis. Antiplatelet or anticoagulant agents may affect atherosclerosis, so further research is needed for investigation. Finally, serum LDL-C levels and body mass index, which have a significant effect on THR and atherosclerosis, were not identified. Although serum LDL-C level is an established risk factor for coronary heart disease, the association between LDL-C and cerebral atherosclerosis is controversial (41). In a retrospective analysis of patients with ischemic stroke or transient ischemic attack, an elevated level of serum THR, but not LDL-C, was associated with large artery atherosclerotic stroke in close agreement with the results of our study (41). In addition, THR was shown a better predictor of LDL particle size than LDL-C in nondiabetic patients (18, 37, 42, 43). Therefore, LDL-C levels are less likely to affect the association between THR and ICAS. A firm conclusion could not be drawn based on results from the present study; thus, further prospective observations conducted on the general population are necessary to validate the results.

## Conclusion

THR was independently correlated with ICAS. Conversely, THR did not show any significant value for diagnosis of other cerebrovascular pathologies. Based on the study results, THR can be used as a feasible, efficient, routine, immediately obtainable, and inexpensive biomarker for ICAS. Because various treatment strategies necessary to reduce the risk of ischemic stroke depend on different cerebrovascular pathologies, further studies are needed, and nonstatin lipid-lowering agents for increasing HDL-C and reducing TG could be a promising treatment strategy for prevention of ICAS-related stroke.

## Data Availability Statement

All datasets generated for this study are included in the article/supplementary material.

## Ethics Statement

The studies involving human participants were reviewed and approved by The Institutional Review Board (IRB) of CHA Bundang Medical Center approved the study (IRB no. BD-2010-083). The patients/participants provided their written informed consent to participate in this study.

## Author Contributions

M-HW and KOL: conception, design, drafting of manuscript, acquisition of data, final approval of manuscript. DC: design, acquisition of data, revision of manuscript, final approval of manuscript. J-WC: design, acquisition of data, revision of manuscript, final approval of manuscript. S-HK: design, acquisition of data, interpretation of data, revision of manuscript, final approval of manuscript. S-HO: conception, design, analysis and interpretation of data, drafting of the manuscript, revision of manuscript, final approval of manuscript. All authors contributed to the article and approved the submitted version.

## Conflict of Interest

The authors declare that the research was conducted in the absence of any commercial or financial relationships that could be construed as a potential conflict of interest.
